# 

**Published:** 1877-01-20

**Authors:** 


☞ All communications relating to the business of THE RECORD, for the year 1877, must be addressed to
DR. R. C. WORD,
Business Manager, South. Med. Rec.,
Atlanta, Ga.

☞ Brief and practical communications are solicited on all subjects pertaining to medicine, also reports of cases in practice.

☞ Send money by check, postal order, or registered letter.

☞ Write your name, post-office, county and State plainly.

☞ Our list of co-laborers will be published in the February number.

### RECEIPTS.

The following names are *receipted* on renewals for 1877, up to the time of going to press:

L. D. Johnson, John Hardeman, Preston Bond, A. D. Pound for six months, J. H. Howard, A. H. Sellers, W. J. Lee, E. Y. Flemming, John O'Brien, G. B. Battle, J. L. Martin, E. W. Hunter, C. D. Satman, A. P. Harris, J. W. Gilbert, Robert Kells, B. F. Darnell, D. W. Delbridge, S. M. Hogan, W. K. Jones, John A. Hanks, J. H. Henry, J. P. Olliver, C. W. McDaniel, L. W. Mobley, F. B. Calling, A. G. Smythe, L. J. Brownlee, G. H. Thompson, F. De'Lee, J. B. Baily, Lanier & Cabe, W. N. Ames, G. A. Dyer, James Underwood, John A. Field, J. E. Terrell, C. C. Jones, E. N. Cushing, John Riches, E. H. Hurst, J. H. Keer, Wm. Goodrich, J. Alexander, W. F. Grasham, A. A. Stanley, J. M. Stansell, R. Fowler, Charles E. Ward, E. R. Young, M. T. Anderson, R. R. Huie, J. W. Butts, Wm. Palmer, J. C. Moody, J. D. Moon, W. E. Brock. K. H. Davis, A. H. Randall, W. W. Mahan Q. J. Mathews, Fred. Savage.

Dr. Powell requests me to say that receipts on our old accounts will be sent by mail.

R. C. WORD,
Business Manager.

